# Insight on automated lesion delineation methods for PET data

**DOI:** 10.1186/s13550-014-0069-8

**Published:** 2014-12-14

**Authors:** Azadeh Firouzian, Matthew D Kelly, Jérôme M Declerck

**Affiliations:** 1 Siemens plc, Healthcare Sector, Molecular Imaging, 23/38 Hythe Bridge Street, Oxford OX1 2EP, UK

**Keywords:** PET, Delineation, Oncology, Radiotherapy, Tumour volume, Reconstruction

## Abstract

**Background:**

Defining tumour volume for treatment response and radiotherapy planning is challenging and prone to inter- and intra-observer variability. Various automated tumour delineation methods have been proposed in the literature, each having abilities and limitations. Therefore, there is a need to provide clinicians with practical information on delineation method selection.

**Methods:**

Six different automated positron emission tomography (PET) delineation methods were evaluated and compared using National Electrical Manufacturer Association image quality (NEMA IQ) phantom data and three in-house synthetic phantoms with clinically relevant lesion shapes including spheres with necrotic core and irregular shapes. The impact of different contrast ratios, emission counts, realisations and reconstruction algorithms on delineation performance was also studied using similarity index (SI) and percentage volume error (%VE) as performance measures.

**Results:**

With the NEMA IQ phantom, contrast thresholding (CT) performed best on average for all sphere sizes and parameter settings (SI = 0.83; %VE = 5.65% ± 24.34%). Adaptive thresholding at 40% (AT40) was the next best method and required no prior parameter tuning (SI = 0.78; %VE = 23.22% ± 70.83%). When using SUV harmonisation filtering prior to delineation (EQ.PET), AT40 remains the best method without prior parameter tuning (SI = 0.81; %VE = 11.39% ± 85.28%).

For necrotic core spheres and irregular shapes of the synthetic phantoms, CT remained the best performing method (SI = 0.83; %VE = 26.31% ± 38.26% and SI = 0.62; %VE = 24.52% ± 46.89%, respectively). The second best method was fuzzy locally adaptive Bayesian (FLAB) (SI = 0.83; %VE = 29.51% ± 81.79%) for necrotic core sphere and AT40 (SI = 0.58; %VE = 25.11% ± 32.41%) for irregular shapes. When using EQ.PET prior to delineation, AT40 was the best performing method without prior parameter tuning for both necrotic core (SI = 0.83; %VE = 27.98% ± 59.58%) and complex shapes phantoms (SI = 0.61; %VE = 14.83% ± 49.39%).

**Conclusions:**

CT and AT40/AT50 are recommended for all lesion sizes and contrasts. Overall, considering background uptake information improves PET delineation accuracy. Applying EQ.PET prior to delineation improves accuracy and reduces coefficient of variation (CV) across different reconstructions and acquisitions.

## Background

^18^F-2-Fluoro-2-deoxy-D-glucose (^18^F-FDG) positron emission tomography (PET) provides information about the metabolic activities of tissue cells and is widely used in cancer management. Cancer cells have increased cellular metabolism of glucose and therefore their ^18^F-FDG uptake is typically higher than healthy tissue cells [1]. Based on this uptake, the location and extent of cancerous tumours can be determined to support tumour staging, evaluation of treatment response and radiotherapy planning. FDG PET provides complementary information to anatomical imaging modalities such as computed tomography to aid the differentiation between healthy and malignant tissue. It also allows determination of metabolic tumour volume (MTV) and consequently total lesion glycolysis (TLG) [2] which have shown prognostic and predictive value in oncology [3],[4]. PET can also be combined with computed tomography in radiation treatment planning for more accurate gross tumour volume (GTV) definition [5]. In addition, ^18^F-FDG PET can show metabolic inhomogeneity inside the tumour which can be used to identify areas in the tumour which may benefit from additional radiation [6].

Currently, tumour volume delineation is typically performed manually by visual interpretation of PET or computed tomography images, which is prone to inter- and intra-observer variability [7]-[9]. The relatively low spatial resolution of PET and noise contribute to this variation by making the lesion boundaries less well defined. Numerous studies have proposed a variety of automatic delineation methods to overcome the subjectivity of tumour volume delineation, with the most commonly used being thresholding a volume of interest (VOI) including the tumour by 40% or 50% of maximum standard uptake value (SUV_max_) within the VOI. This method is used in routine clinical practice and provides clinical benefits [2],[10]. Other automatic PET delineation methods include contrast-oriented [11], gradient-based [12], adaptive thresholding [10], background-subtracted relative-threshold level [13] and statistical modelling (FLAB) [14] and their performance have been compared in several studies [15]-[18]. These studies used either clinical data with histology, computed tomography or manually drawn contours as ground truth or phantom or simulation data with known ground truth but reconstructed with a single reconstruction protocol. They showed that the performance of a delineation method depends on imaging parameters (i.e. reconstruction settings, image noise level, tumour characteristics, contrast) and you need to choose the suitable delineation method accordingly to get the optimum results.

In this study, we used National Electrical Manufacturer Association (NEMA) image quality (IQ) [19] phantom data acquired with a range of contrasts and emission counts and reconstructed with a range of reconstruction protocols, using the manufactured sphere sizes as ground truth. Additionally more clinically relevant, heterogeneous and irregularly shaped lesions were studied using synthetic phantoms created in-house and reconstructed with the same set of contrasts, emission counts and reconstruction protocols as for the NEMA IQ phantom. Using phantom data enabled us to validate against a real ground truth and therefore assess the performance of the methods more precisely and objectively. We used this phantom data to evaluate a range of automatic tumour delineation methods for PET data which use SUV as the classification feature (first-order feature), independently from the inventors of the methods themselves.

The performance of automatic PET delineation methods based on SUV will be affected by factors known to impact SUV such as scanner type and reconstruction parameters [20]. However, there have been limited studies that considered the impact of these factors on tumour volume assessment [21]. Therefore, an additional objective of this study was to assess quantitatively the effect of those factors on the automatic delineation methods and compare their performance before and after correction for differences in recovery [22].

In practice, it is very important to consider the clinical objective when selecting the delineation method. For example, in radiotherapy, the aim is to delineate the extent of the tumour as precisely as possible to avoid unnecessary irradiation of healthy tissue. As such, the mean accuracy of the method would be the key performance measure. For tumour response assessment, the objective is accurate determination of change in metabolic volume. As such, it may be preferred to use a method which is consistent across all reconstructions and acquisitions accepting any tendency to over- or under-estimate the true volume. The objective of this study is to provide information to inform the selection of delineation method.

## Methods

Six different automatic PET delineation methods were evaluated using NEMA IQ phantom and three additional synthetic phantom data sets created in-house.

### Physical phantom

The NEMA IQ phantom was prepared with an ^18^F solution to produce a background activity concentration of 5.2 kBq/ml. The six spheres (10-, 13-, 17-, 22-, 28- and 37-mm diameter) were filled to produce a hot sphere-to-background ratio of either 4:1 or 8:1. The phantom was placed in the 64 slice Biograph mCT scanner (Siemens Healthcare, Erlangen, Germany) such that the plane through the centre of the spheres was aligned with the centre of the PET axial field of view and the centre of the lung insert was aligned with the centre of the transaxial field of view. For each concentration ratio, a 1-h listmode acquisition was performed. Using an electrocardiogram (ECG) simulator, the listmode data was gated and divided into ten replicate sinograms for each of 3.0 × 10^7^ and 6.0 × 10^7^ net true coincidences. Each replicate was reconstructed with four different reconstruction protocols representing a range of clinically relevant configurations; namely: 3-dimensional ordinary Poisson ordered subset expectation maximisation (3D OP-OSEM) with 3 iterations, 24 subsets and 5-mm full width at half maximum (FWHM) Gaussian post filter; 3D OP-OSEM + time of flight (TOF) with 2 iterations, 21 subsets, 2-mm FWHM Gaussian post filter; 3D OP-OSEM + point spread function (PSF) with 3 iterations, 24 subsets and 2-mm FWHM Gaussian post filter; 3D PSF + TOF with 2 iterations, 21 subsets and 2-mm FWHM Gaussian post filter. Each reconstruction protocol was performed on both a 200 × 200 and 400 × 400 matrix, giving voxel dimensions of 4.073 × 4.073 mm with a 2.027-mm slice thickness, and 2.036 × 2.036 mm with a 2.027-mm slice thickness, respectively. In total, 6 (spheres) × 2 (contrasts) × 4 (reconstruction protocols) × 3 (emission counts) × 10 (replicates) × 2 (matrix sizes) were acquired, making a total of 2,880 objects to be delineated.

### Synthetic phantoms

**Figure 1:**
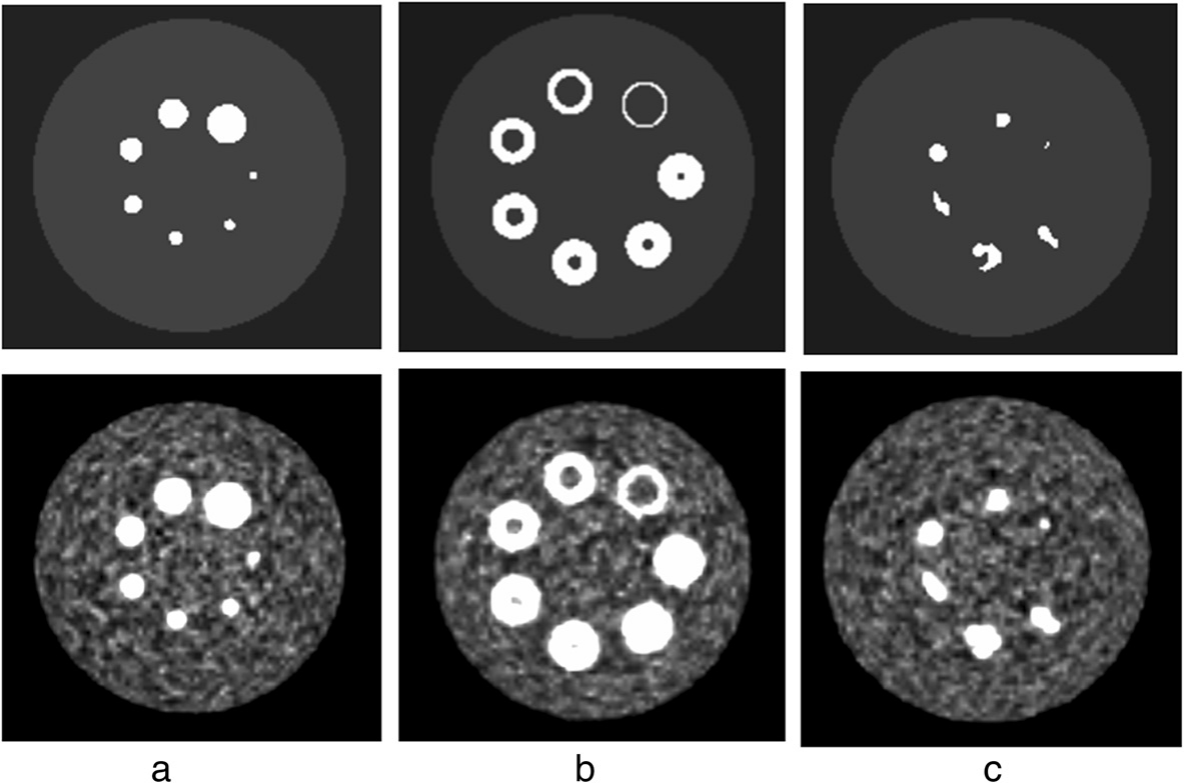
**The three synthetic phantom configurations before (first row) and after reconstruction (second row).** The presented reconstructed examples have a contrast of 8 to 1, 60-M emission counts and is reconstructed with OPTOF (2 iterations, 21 subsets, 2-mm post filter size) and 200 × 200 pixels matrix size; **a)** including 7 spheres with diameters of 7, 10, 13, 17, 22, 28 and 37 mm, **b)** including 7 spheres with necrotic core, outer diameters of 42 mm and inner diameters of 7, 10, 13, 17, 22, 28 and 37 mm, **c)** including 6 different lesion shapes extracted from real patient data.

**Figure 2:**
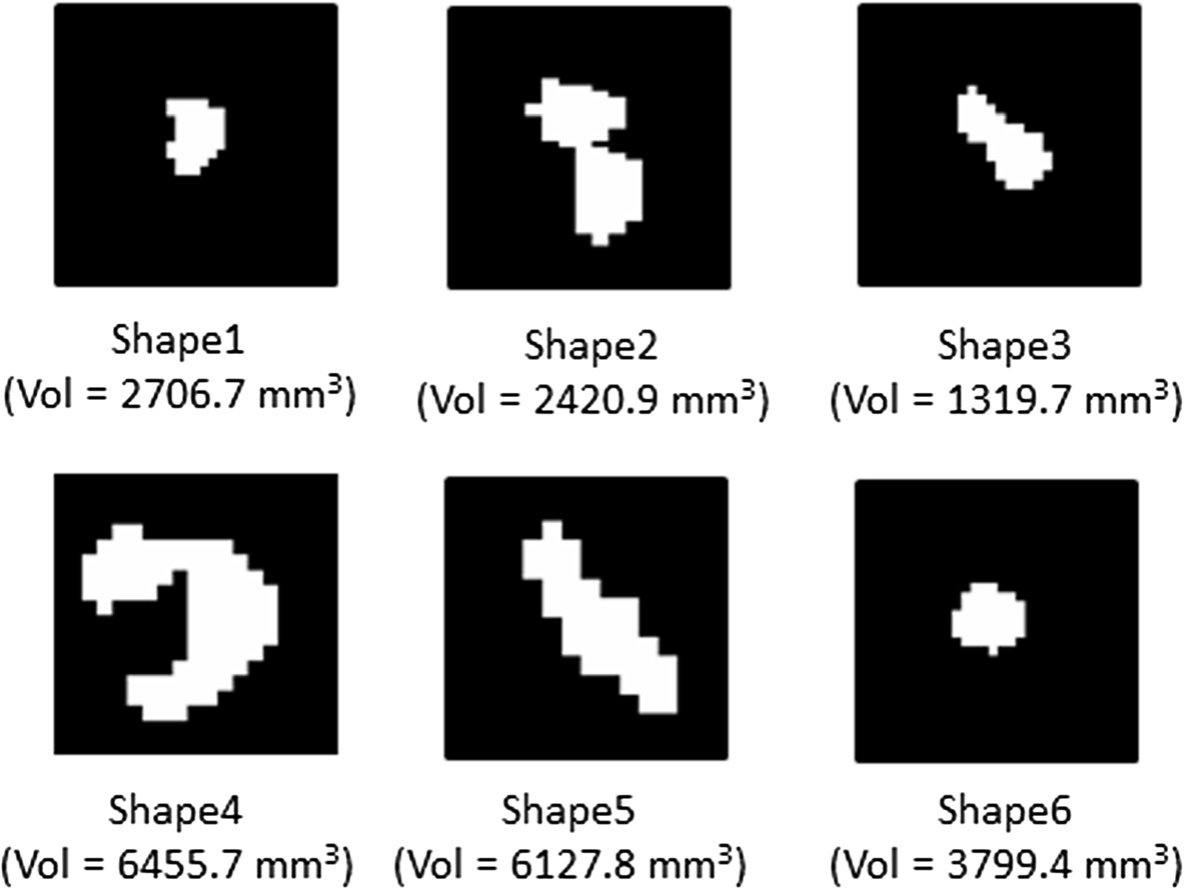
**Original binary masks (axial view) of example lesion shapes created from real patient data.** Volume (Vol) of each lesion is shown below each mask.

In addition to the physical phantom, three different synthetic phantoms were designed to evaluate the performance of the automated PET delineation methods with more complex lesion shapes. All three synthetic phantoms have a cylindrical body (diameter = 300 mm, length = 221 mm). In the first phantom, seven spheres were placed radially around the central transaxial slice with diameters of 7, 10, 13, 17, 22, 28 and 37 mm (Figure 1a). A similar configuration was used for the second phantom (Figure 1b) but with all the spheres having a diameter of 42 mm and including necrotic cores of the same diameters as the spheres placed in the first phantom. The third phantom (Figure 1c) included six irregular shapes (Figure 2) selected from real patient data. Only the contours were used in the simulation, and the uptake was assumed constant within the contour of the lesion, which was digitised to match the image matrix. PET equivalent data for these synthetic phantoms were simulated using in-house PET simulator software that forward projects the input image using the geometry of the Siemens mCT (Siemens Healthcare, Erlangen, Germany) [23], including effects of detector sensitivity, attenuation, scatter, randoms and Poisson noise. The simulations included 3.0 × 10^7^ or 6.0 × 10^7^ true events and 5.0 × 10^7^ or 10.0 × 10^7^ random events, respectively, representing a typical patient acquisition (weight of 75 kg) with approximately 2.5 or 5 min per bed position and 10 mCi injection (scanned 45 min after injection). Ten replicate sinograms were generated for each of 3.0 × 10^7^ and 6.0 × 10^7^ net true coincidences similar to the real phantom. The generated sinograms and corresponding μ-maps for attenuation correction were reconstructed with the same reconstruction algorithms available on the commercial system. The same reconstruction parameters were used for the synthetic phantom as for the physical NEMA IQ to allow direct comparison.

### EQ.PET filter

SUV is intended to reduce the effect of patient size and body composition relative to the injected dose of radiotracer and making it possible to compare between different studies and patients [4],[24]. However, there are other factors that can introduce bias to the quantification process, such as reconstruction protocol. An additional reconstruction-specific Gaussian filter (EQ.PET filter) was proposed to overcome this bias [22]. The filter size for a given reconstruction protocol is determined by minimising the root mean squared error (RMSE) between the recovery coefficients (RCs) of a NEMA IQ phantom reconstructed with that protocol and those of a common reference [10]. The Gaussian filters aligning the recovery coefficients for each reconstruction protocol to the reference have been applied prior to automatic delineation and the results compared to those generated with no additional filtering. The EQ.PET filter sizes for OP (with 3 iterations, 24 subsets and 5-mm FWHM Gaussian post filter), OP + TOF (with 2 iterations, 21 subsets, 2-mm FWHM Gaussian post filter), PSF (with 3 iterations, 24 subsets and 2-mm FWHM Gaussian post filter) and PSF + TOF (with 2 iterations, 21 subsets and 2-mm FWHM Gaussian post filter) were 4.76, 7.35, 6.52 and 7.41, respectively.

### Automated PET delineation methods

For each phantom, each object being delineated was enclosed in a bounding VOI containing no other objects. Six different automatic PET delineation methods were applied to the VOIs, with and without prior EQ.PET filtering, including:

 Thresholding at 40% SUV_max_ (T40): delineates all voxels with SUVs above or equal to 40% of the maximum SUV inside the selected VOI.

 Thresholding at 50% SUV_max_ (T50): delineates all voxels with SUVs above or equal to 50% of the maximum SUV inside the selected VOI.

 Contrast thresholding (CT) [11]: uses an optimal threshold (T) for the data inside the selected VOI that was calculated as follows:

(1) $$T=a\times mSUV_{70}+b\times BG$$

where *mSUV*_*70*_ is the mean SUV in the region generated by thresholding the VOI at 70% of SUV_max_ and *BG* is the mean SUV in a background region. Parameters *a* and *b* are calculated using a set of NEMA IQ phantom acquisitions to determine a regression function that best represents the relationship between the optimal threshold for that phantom and its *mSUV*_*70*_ and *BG*. In this study, we performed the regression analysis using the NEMA IQ phantom data described in the Physical phantom section.

 Adaptive thresholding at 40% SUV_max_ (AT40) [10]: adapts the threshold value inside the selected VOI relative to mean background SUV (*BG*):

(2) $$T=0.4\;\times \left(SUV_{\max}-BG\right)+BG$$

Adaptive thresholding at 50% SUV_max_ (AT50) [10]: adapts the threshold value inside the selected VOI relative mean background SUV:

(3) $$T=0.5\;\times \left(SUV_{\max}-BG\right)+BG$$

Fuzzy locally adaptive Bayesian (FLAB) [14]: an unsupervised statistical method using a fuzzy model within the Bayesian framework. It allows coexistence of voxels belonging to one of the two hard classes and voxels belonging to a ‘fuzzy level’ inside the selected VOI.

For methods which required background uptake information, a spherical background region (20 voxels in diameter) was manually positioned in the body of the phantom away from any spherical inserts or synthetic lesions. All the above methods except FLAB have been developed in house. The FLAB implementation was acquired from the inventors directly and was applied without any modifications in the method itself or its parameter settings.

### Validation

Given the true position and size of phantom spheres is known for the NEMA IQ phantom, ground truth was created on the PET data by fitting a sphere of the appropriate volume to each of the phantom spheres to create a binary mask. The VOIs generated with the automated methods described above were validated against this ground truth. For the synthetic phantoms, the ground truths were the binary masks used as input to the PET simulator.

Two evaluation measures were used to measure the agreement between the ground truth and the delineated lesions: similarity index (SI) (Equation 4) and percentage volume error (%VE) (Equation 5). These were calculated as follows:

(4) $$SI=\frac{2\;\left(GT\cap \mathrm{PET}\right)}{\left(\left|GT\right|+\left|\mathrm{PET}\right|\right)}$$

(5) $$\%VE=\;\left(\frac{{Vol}_{\mathrm{PET}}-\;{Vol}_{\mathrm{true}}}{{Vol}_{\mathrm{true}}}\right)\times 100$$

where *GT* is the number of voxels in the ground truth binary mask, *PET* is the number of voxels in the delineated binary mask produced by the delineation method, *Vol*_*PET*_ is the volume of the automatically delineated sphere and *Vol*_*true*_ is the mathematically calculated volume in case of the NEMA IQ phantom and the initial mask volume for the synthetic phantoms.

## Results and discussion

Automatically delineated VOIs from the NEMA IQ phantom and the synthetic phantoms were compared against ground truth and SI and %VE were calculated for all lesion sizes, clinically relevant reconstruction protocols, contrasts and emission counts. The results are presented with and without applying prior EQ.PET filtering.

### Results for NEMA IQ phantom

**Figure 3:**
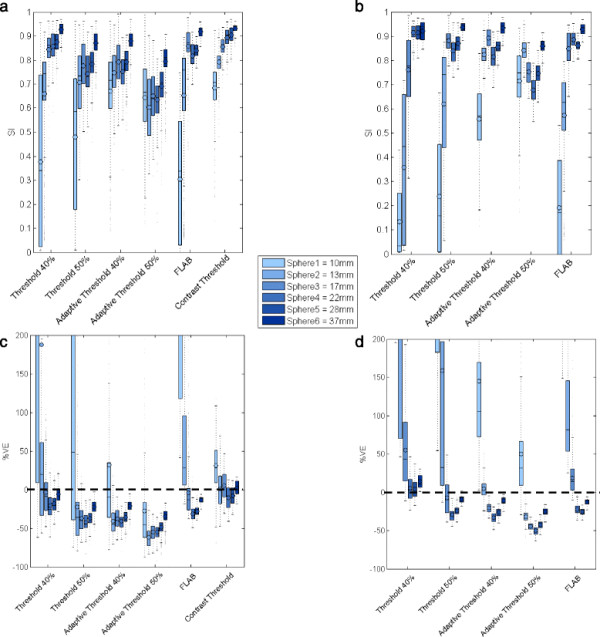
**SI (a, b) and %VE (c, d) for all methods based on validation on NEMA IQ phantom.** For each method a group of 6 boxplots are presented, each belonging to one sphere size, with (b, d) and without (a, c) prior filtering. Each boxplot represents the distribution (mean and quartiles) of validation results from all clinical reconstructions. In case of %VE, the y-axis range has been limited to −100 to 200 for better readability.

Figure 3 shows the validation results using SI (3a, 3b) and %VE (3c, 3d) for each delineation method, across all clinical reconstructions and sphere sizes in the NEMA IQ phantom with (3a, 3c) and without (3b, 3d) EQ.PET filtering. Ideally, SI should be 1 and %VE should be 0%. Consistency of volume delineation across all reconstructions and acquisitions was measured using the coefficient of variation (CV) of the segmented volumes:

(6) $$CV=\;\frac{SD}{\mathrm{mean}}$$

where *mean* is the mean delineated volume for a set of lesions and *SD* is the corresponding standard deviation. Lower CV values represent higher consistency of the delineated volumes across different reconstructions and acquisitions. Across all spheres sizes, CT had the highest mean SI (average over all spheres is 0.83) and lowest %VE (5.56% ± 24.34%; mean ± SD). Given the dependence of the methods’ performance on lesion size, the data was divided into two size groups, large (17, 22, 28 and 37 mm) and small (10 and 13 mm), and average values were calculated for each group. For small spheres, only those methods explicitly considering background uptake (CT, AT40 and AT50) produced sensible delineations, whilst the other methods frequently overestimated the lesion volumes, including a large proportion of background region in the delineation (Figure 3). For threshold-based methods, considering background uptake reduced the inclusion of background by ensuring the threshold value used for delineation was above the mean background value, even for low contrast lesions. Effect of lesion contrast and number of emission counts were also investigated. For small lesions, higher contrast values improved SI, %VE and CV; increased emission counts slightly improved SI and %VE but had no effect on CV. For large lesions, increasing contrast or emission counts slightly improved SI and %VE but did not have significant effect on CV. Whilst increasing counts would be expected to reduce the variation across the ten replicates for a given reconstruction protocol, the variation due to differences in reconstruction protocol dominates this effect resulting in minimal impact on CV.

**Figure 4:**
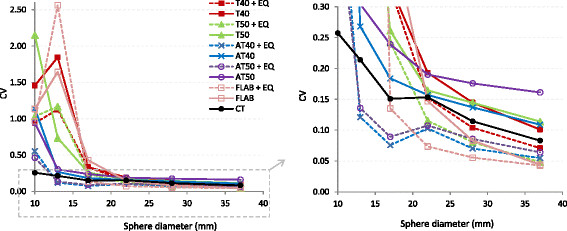
**CV of delineated volumes on NEMA IQ phantom data across different reconstructions and acquisitions.** CV values are presented with respect to lesion size for all methods with (dashed lines) and without (solid lines) prior EQ.PET filtering (left). A zoomed version of the plot over a closer range of CV values is shown on the right.

Following application of the appropriate EQ.PET filter, AT40 had the highest mean SI (0.81) and lowest %VE (11.39% ± 85.28%); however, this SI remains slightly lower and the %VE slightly higher than those of the unfiltered CT method (Figure 3). EQ.PET filtering was not applied to CT since the method parameters were already optimised for each reconstruction. The consistency of the delineated volume across reconstructions improved using EQ.PET filtering: mean CV was reduced approximately 1.6-fold for all sphere sizes and delineation methods. The CV values calculated across all reconstructions and acquisitions for each delineation method, with and without EQ.PET filtering, are shown in Figure 4. For large lesions, the average CV was 0.2 for all delineation methods, whereas for small lesions the CV could reach more than 1. After applying EQ.PET, CV decreased on average to less than 0.1 for large lesions and 0.9 for small lesions. Filtering also increased accuracy (SI) for large spheres from 0.81 to 0.85 but reduced accuracy for small spheres from 0.59 to 0.49.

As it was presented in the results above, effect of applying EQ.PET filter can be different on large and small lesions. The filtering reduces noise in the image and makes it more homogeneous but also reduces contrast between foreground and background. Homogeneity contributes to consistency across all reconstructions and acquisitions by making these images more similar to each other in terms of SUVs. It can also suppress local maxima in background regions, preventing their inclusion in the delineated volume, which can occur with low contrast lesions. EQ.PET typically improved accuracy for large lesions; for example, the AT40 mean %VE decreased from −34.27% to −22.65% for large lesions. For small lesions, EQ.PET typically reduced delineation accuracy; for example, the AT40 mean %VE increased from −1.14% to 79.48% for small lesions. Small lesions often have low image contrast and by further reducing the contrast with EQ.PET, background regions are more likely to be included in the delineation.

### Results for synthetic phantoms

The results from the first synthetic phantom with NEMA size spheres are in line with those from the physical NEMA IQ phantom data as we observed that CT also showed the highest SI (0.73). Similarly to what was done for the analysis of the physical NEMA IQ phantom results, the data was divided into two groups of large (17, 22, 28 and 37 mm) and small (7, 10 and 13 mm) spheres. Adding an extra small sphere (7 mm) to the phantom decreased the accuracy for delineating small spheres for all methods with respect to the physical phantom. For example, CT accuracy (SI) for small spheres decreased from 0.73 to 0.54, although it remained the same as the physical phantom for large spheres. After applying the EQ.PET filter, AT40 had the highest SI (0.71) and the mean CV improved approximately 1.6-fold for all sphere sizes and delineation methods. The accuracy (SI) for small spheres decreased from 0.43 to 0.38 and for large spheres increased from 0.80 to 0.84. The effect of contrast and number of emission counts was similar to the physical phantom.

**Figure 5:**
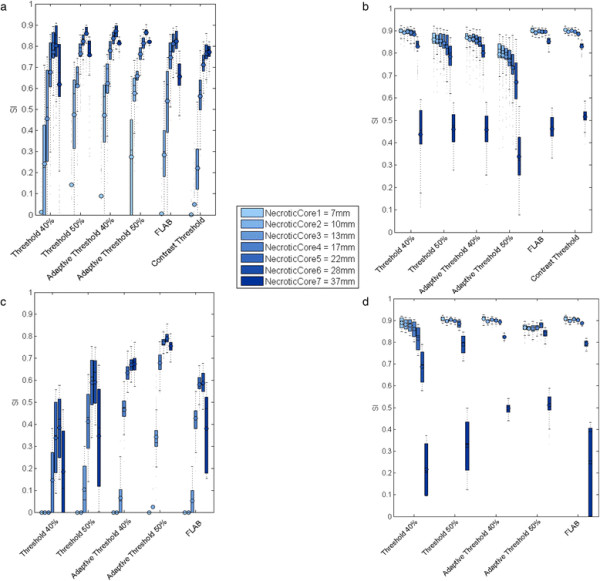
**SI based on the synthetic phantom with necrotic core spheres.** Results are presented for necrotic core **(a, c)** and non-necrotic layer **(b, d)** for all methods. The results are presented with (c, d) and without (a, b) prior EQ.PET filtering. For each method, a group of 7 boxplots are presented, each belonging to one necrotic core size. Each boxplot represents the distribution (mean and quartiles) of validation results from all clinical reconstructions.

**Figure 6:**
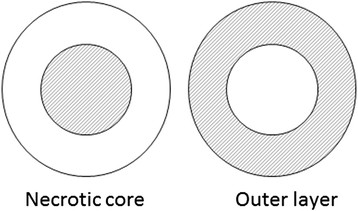
**Schematic drawing of the necrotic core spheres in the synthetic phantom.** The performance of each method has been validated based on the ability of the method to delineate either the inner core or outer layer, shaded areas in the left and right diagrams, respectively.

**Figure 7:**
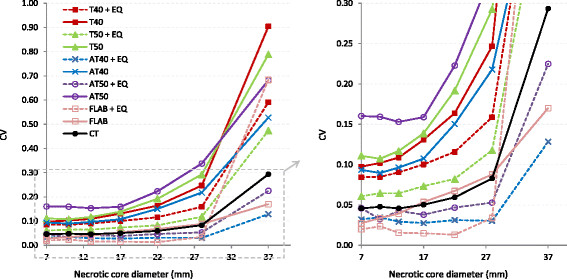
**CV of delineated volumes from the synthetic phantom with necrotic core spheres.** The results are presented across different reconstructions and acquisitions for all delineation methods with respect to necrotic core size with (dashed lines) and without (solid lines) prior EQ.PET filtering (left). The zoomed version of the plot is shown on the right

Figure 5 shows the results (SI) from the synthetic phantom with necrotic core spheres with (5c, 5d) and without (5a, 5b) prior EQ.PET filtering. The validation has been performed based on both the necrotic core and the outer layer (Figure 6) since focusing only on the outer layer may not reflect the ability of the method to detect the necrotic core. On average, when validating on the outer layer, CT has the highest SI (0.83) with %VE of 26.31% ± 38.26% and after applying EQ.PET filter, AT40 has the highest SI (0.83) with %VE of 27.98% ± 59.58%. In addition, similarly to the physical NEMA IQ phantom results, filtering reduced mean CV approximately 2-fold for small cores and approximately 2.4-fold for large cores for all delineation methods (Figure 7). For large necrotic cores, filtering had negligible effect on accuracy (SI) and for small necrotic cores, the accuracy slightly increased from 0.86 to 0.89. When validating on the necrotic core, AT50 has the highest SI without (0.68; %VE = 35.26% ± 56.45%) and with (0.48; %VE = 57.5% ± 31.92%) prior EQ.PET filtering. Furthermore, filtering reduced the mean accuracy (SI) for small cores from 0.36 to 0.04 and from 0.78 to 0.52 for large cores. CV increased approximately 1.5-fold for the small cores and approximately 1.6-fold for the large ones.

**Figure 8:**
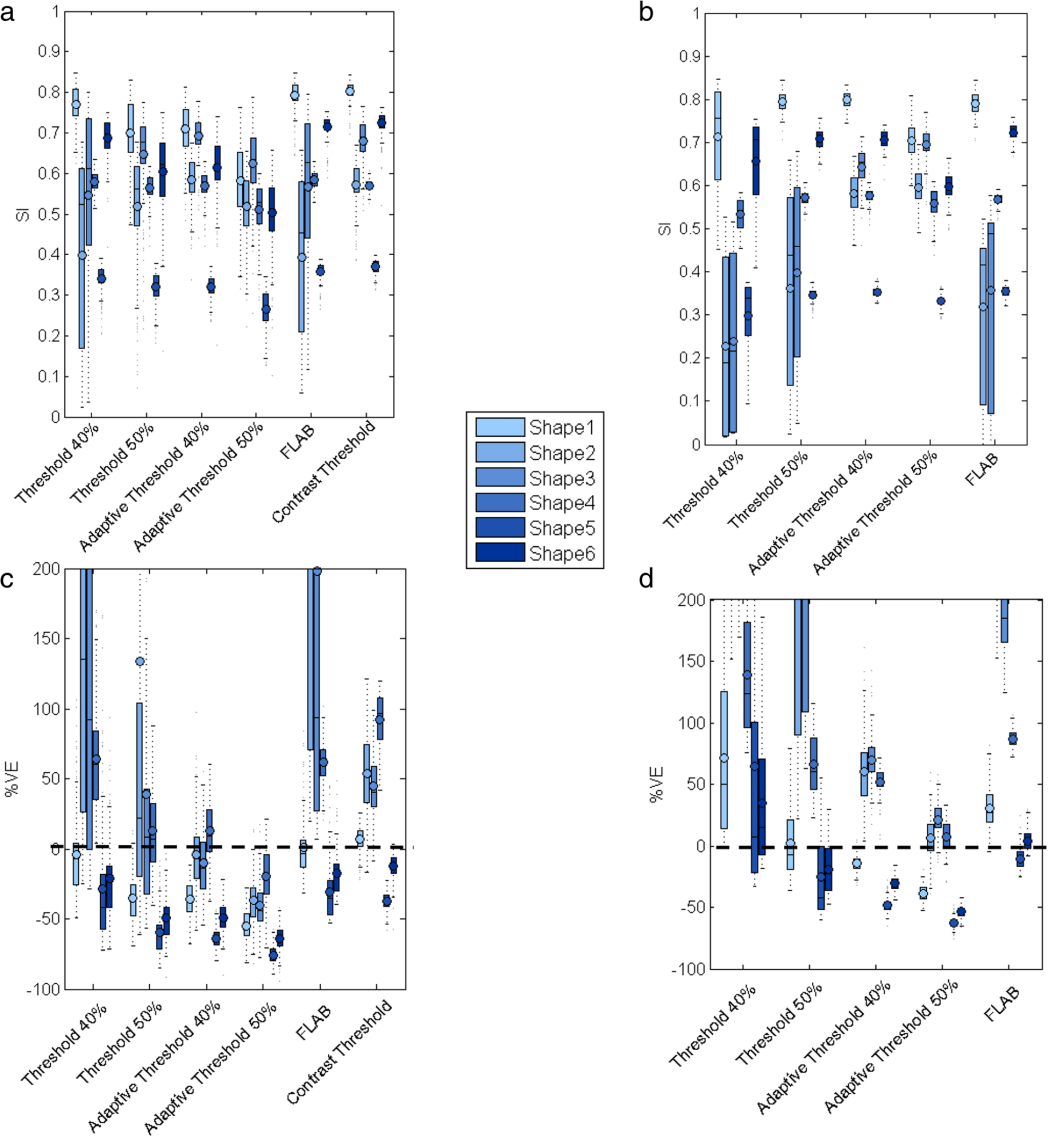
**SI (a, b) and %VE (c, d) for all methods using synthetic phantom data with irregular shapes.** For each method a group of 6 boxplots are presented, each belonging to one shape with (b, d) and without (a, c) prior EQ.PET filtering. Each boxplot includes the validation results from all clinical reconstructions. In case of %VE, the y-axis range has been limited to −100 to 200 for better readability.

The validation results on the phantom with irregular shapes show that CT has the highest SI (0.62; %VE = 24.52% ± 46.89%) for all shapes (Figure 8). After applying EQ.PET filtering AT40 has the highest SI (0.61; %VE = 14.83% ± 49.39%) which is a similar SI as CT without prior filtering but improved %VE. Prior filtering slightly improved overall mean accuracy but CV reduced approximately 1.4-fold. The more the shapes deviate from a sphere, the less accurate the delineation results. The algorithms capable of delineating small lesions performed better on these shapes since they were able to delineate small details better.

### Practical insights on selecting a lesion delineation method

**Figure 9:**
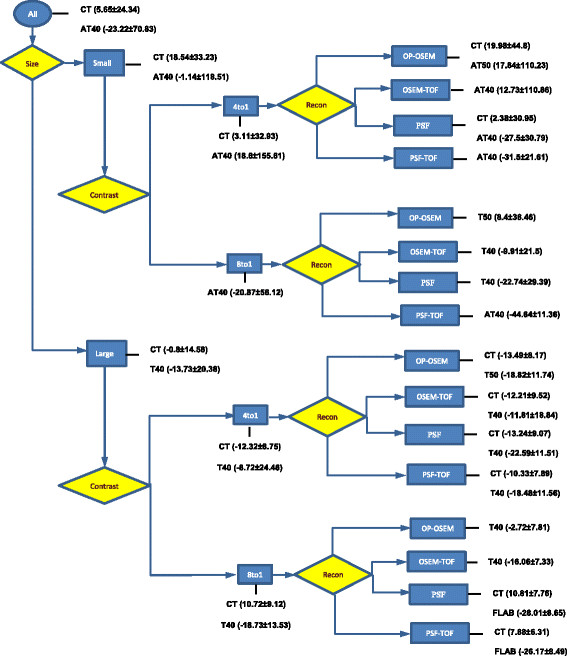
**Flow chart summarising methods’ performance for each parameter setting on NEMA IQ data.** The parameters are shown as diamonds and are ordered from high to low impact from left to right. The options for each parameter are presented in rectangles and the best performing method this type of data is presented alongside these rectangles. The methods are ordered according to accuracy and the corresponding %VE ± SD are provided between brackets.

To produce guidelines to assist selection of the best method for a specific data type, the effects of different data parameters including lesion size, lesion contrast, reconstruction protocol and number of emission counts have been studied. From our results, lesion size and contrast had the most impact on method selection whereas reconstruction parameters and number of emission counts had a lower impact but they can be used to fine tune the accuracy. A summary of the methods’ performance on NEMA IQ phantom data, based on different parameters, is shown in Figure 9. This diagram provides an overview of the relative performance of the best performing methods for different situations and provides an indication of the level of accuracy you can expect in each situation. The parameters which have impact on the algorithm performance are shown as diamonds and option for each parameter is indicated next to them. Next to each option, the performance of the selected method is presented in terms of %VE ± SD in brackets. Depending on the type of data and application, clinicians can use this flowchart to aid their selection of the most appropriate method.

Based on the results presented in this study, CT is the best performing method for all lesion types but its utility is limited by the requirement for prior parameter tuning using phantom data which might not be available in all clinical sites. For the experiments performed on the real NEMA IQ phantom data, the method parameters (a and b) were calculated on the same data as used for the assessment, which could result in an overly optimistic assessment of the performance of the method. However, this was not the case for the simulated phantom data and the performance observed with these datasets was in agreement with the real phantom data. Furthermore, whilst these phantom-tuned parameters perform well on the more complex lesion shapes used in this study, we have not evaluated their performance on heterogeneous lesions (beyond those with a necrotic core), or lesions in a heterogeneous background. AT40 or AT50 show only marginally reduced performance and require no prior tuning. AT performs especially well on small lesions even with low contrast. CT and AT are both using background uptake information which makes them more capable of handling small low contrast lesions. For large high contrast lesions, most methods perform reasonable but with different accuracy levels. Depending on the application and availability, clinicians can decide whether they would like to opt for slightly higher accuracy with prior tuning or not. These findings are consistent with a previous study comparing different PET delineation methods (except FLAB) where they found AT40 and CT are the best performing methods for assessing lesion sizes in comparison to thresholding, relative thresholding [13], absolute SUV and gradient-based watershed [12] method [15]. We did not observe the improved performance of FLAB relative to other methods that has been reported by the inventors of the method in a previous study [25]. This may be because the FLAB implementation we used had not been optimised for the reconstruction protocols or types of objects used in this study.

For methods requiring information on background uptake (CT and AT), the background region needs to be defined by the user which might introduce some variations in the results. For this study, it was not the case since the phantom body is a homogeneous structure, but in clinical practice, the background region needs to be selected carefully. For FLAB, the background uptake is estimated from the initial VOI and therefore the results might vary based on different VOI selections. FLAB allows the user to tune various parameters to the data, but in this study, we avoided user interference to minimise any bias in our results. The VOI definition for thresholding methods does not introduce variations in the lesion size but might include false positives if it is too large.

To give a more clinical context to the results, the best performing scenario for small spheres in the NEMA phantom, PSF reconstruction with CT gives a mean %VE of 2.4% ± 31.0%. Of relevance to radiotherapy planning, this translates to a mean volume error of 19.0 ml ± 66.0 ml. For MTV-based response assessment, this translates to a 95% confidence interval of approximately 61% on the volume measurement. For large spheres, the best scenario would be OP-OSEM with T40 which gives a mean %VE of −2.7% ± 7.8% which is translated to a volume error of −305.4 ± 762.4 ml and translates into confidence interval of 15.3%.

## Conclusions

Considering background uptake information in the delineation process improves accuracy, especially for small, low contrast and heterogeneous lesions. There was less variation in performance for large, high contrast lesions. CT was the most accurate method on average but requires additional parameter tuning that might not be possible at all clinical sites. The accuracy of AT40 was only marginally lower than CT and requires no additional parameter tuning. Amongst the parameters investigated, lesion size and contrast had the biggest impact on the relative performance of the delineation methods evaluated. The variation in delineated volumes for small lesions, even for the simple NEMA spheres, was generally very high (CV greater than 1) and relatively low (CV smaller than 0.5) for large lesions across the different reconstruction methods. Applying EQ.PET filtering to the data prior to delineation reduced the variation in delineated volume for all lesion sizes (e.g., CV less than 0.15 for lesions greater than 10-mm diameter with AT methods). EQ.PET filtering had a variable effect on delineation accuracy, typically improving the performance for large lesions and reducing it for small lesions.

## Abbreviations

PET: positron emission tomography

NEMA IQ: National Electrical Manufacturer Association image quality

SI: similarity index

%VE: percentage volume error

CT: contrast thresholding

AT: adaptive thresholding

CV: coefficient of variation

F-FDG: ^18^F-2-fluoro-2-deoxy-D-glucose

MTV: metabolic tumour volume

TLG: total lesion glycolysis

GTV: gross tumour volume

VOI: volume of interest

SUV: standard uptake value

FLAB: fuzzy locally adaptive Bayesian

ECG: electrocardiogram

FWHM: full width at half maximum

3D OP-OSEM: 3-dimensional ordinary Poisson ordered subset expectation maximisation

TOF: time of flight

PSF: point spread function

RC: recovery coefficients

RMSE: root mean squared error

SD: standard deviation

T: thresholding

## Competing interests

The authors are employed by Siemens Healthcare.

## Authors’ contributions

AF was responsible for implementation of the study, data acquisition, performing the experiments, analysing the results and preparing the manuscript. MK and JD contributed to the study design as well as analysis of the results and reviewing the manuscript. All the authors read and approved the final manuscript.
